# Citric acid as a factor limiting changes in the quality of table eggs during their storage

**DOI:** 10.1016/j.psj.2021.01.018

**Published:** 2021-01-16

**Authors:** Kamil Drabik, Justyna Batkowska, Tomasz Próchniak, Beata Horecka

**Affiliations:** Institute of Biological Basis of Animal Production, University of Life Sciences in Lublin, 20-950 Lublin, Poland

**Keywords:** eggs storage, pores sealing, eggshell structure

## Abstract

The aim of the experiment was to evaluate the potential use of citric acid as a modifier of quality changes in table eggs during their storage. About 780 table hen eggs were collected on the same day. They were numbered individually and placed on trays 30 pcs on each. Control group (CA0) consisted of eggs unmodified with any additional substances. In experimental groups CA10 and CA15, eggshells were sprayed with the aqueous solution of citric acid (10 and 15% concentration, respectively). At the start of the experiment, only quality traits of eggs from the control group were analyzed. The remaining eggs were stored at 14°C and 70% RH (typical storage conditions). Their quality was evaluated after 7, 14, 21, and 28 d. The depth of the air cell, egg weight and specific gravity, traits of shell (permeability, strength, weight, thickness, density), and egg content (pH of yolk and albumen, Haugh units, yolk weight and color) were evaluated each time. The use of citric acid decreased the severity of qualitative changes. Citric acid–treated eggs demonstrated smaller weight loss, shallower air cell, higher structural albumen, less-intensive water diffusion from albumen to yolk indicating the improved resistance of the vitelline membrane. Owing to the fact that citric acid is accepted and recognized as a safe food preservative is a relatively cheap and available substance, it seems that it can be used to inhibit quality changes in table eggs during their storage.

## Introduction

The basic quality of table eggs depends on a variety of factors (genotype, age, rearing system, nutrition, etc.). Regardless of the initial values of the eggs' quality characteristics, negative changes emerge over time. One of the fundamental transformations is the natural weight loss of eggs due to the gaseous exchange (water evaporation) between the egg content and the external environment. This process also affects other egg quality traits, including the air cell depth. These changes are all the more important as they form the basis of the current EU legislation. Commission Regulation (EC) No. 589/2008 (Commission Regulation (EC), 2008) specifies a minimum shelf-life for table eggs at 28 d and a maximum air cell depth of 6 mm. More importantly, the regulation stipulates that the refrigerated storage of eggs is reserved exclusively for final consumers.

Despite the confirmed positive results of cold storage in inhibiting the egg quality deterioration (Jin et al., 2010; Brodacki et al., 2019), this restriction makes it necessary to seek alternative solutions to this problem. Methods inhibiting the quality changes of table eggs may be divided into 2 main groups: the modification of the atmosphere in the storage container and the use of eggshell coating substances.

In case of MAP (modified atmosphere packaging), standard gas mixtures (Rocculi et al., 2009) as well as pure gases in high concentrations (Pasquali et al., 2012; Jin et al., 2019) have already been used for atmosphere modification. Studies carried out by Aygun and Sert (2013) have also shown the viability of extending storage time by means of vacuum packaging.

However, the use of atmospheric modifications requires specialized technological solutions. Therefore, substances coating the eggshell are used much more frequently. So far, the use encompassed both vegetable and mineral oils (Jirangrat et al., 2010; Ryu et al., 2011; Eke et al., 2013; Nongtaodum et al., 2013), chitosan and its emulsions with oils (Torrico et al., 2010, 2014; Jo et al., 2011), propolis (Copur et al., 2008), and many others. In general, eggshell coating results in the reduction of gaseous exchange (water evaporation) from the egg. According to Campo et al. (2000), the gaseous exchange between the egg content and external environment is almost 2 times greater than in reverse direction. Therefore, limiting the shell pores' permeability has the effect of inhibiting the fundamental changes in the eggs' quality during their storage, as confirmed by the aforementioned articles.

Apart from the eggshell becoming sealed by coating, we also considered changing the structure of the shell itself, which should reduce the gas exchange as well. Owing to the high calcium content in the eggshell, the use of acids to seal the shell pores seems plausible. As a result of the reaction between calcium and acid, the surface thickness of the shell will probably decrease. However, the pores should be filled with the products of this reaction. One such acid may be citric acid (*Acidum citricum*) which is a natural component of any living organism, where it plays an important role in the carbohydrates metabolism. In addition, in the ionic form, that is, citrate, it is an important indirect product in the Krebs cycle. As one of the fruit acids, citric acid is mainly found in citrus fruits such as lemons and oranges. However, the chief means of obtaining this preservative is by cultivating *Aspergillus niger* (Dhillon et al., 2013).

Importantly, hitherto studies have shown the effectiveness of citric acid in the disinfection of Japanese quail hatching eggs (He et al., 2020). At the same time, the authors observed a decrease in the thickness of eggshells indicating the occurrence of the reaction described above.

The aim of the experiment was to evaluate the potential use of citric acid as a substance inhibiting negative changes in the quality of table eggs during storage.

## Materials and methods

### Preliminary Experiment

The aim of the preliminary experiment was to establish the effective concentration of the experimental factor (citric acid, CA). The material consisted of hen eggs from Polbar breed, belonging to Polish genetic resources added to the World Watch List for Domestic Animal Diversity by the Food and Agricultural Organization and maintained at the Laura Kaufman Didactic and Research Station of Small Animals, belonging to the University of Life Sciences in Lublin (Poland) (Gryzińska et al., 2015). Fifty eggs were collected on the same day and divided into equal groups. Zero factor was used in the control group (CA0). In other groups (CA5, CA10, CA15, CA20), eggs were sprayed with the citric acid solution at a concentration of 5, 10, 15, and 20%, respectively. All aqueous solutions were prepared using percentage by weight (*w/v*) formula. After preparation, the pH of obtained solutions were measured (average of 3 samples). In particular groups, it amounted to 1.95, 1.80, 1.72, and 1.51, respectively. After natural drying, eggs were placed on trays (10 pcs each) in the blunt-end-up position and stored at room conditions (21°C; 45% of relative humidity) for 28 d.

Weekly changes of the egg weight and air cell depth were recorded. The analyses covered selected quality parameters such as eggshell strength (Instron 55 Mini apparatus), shell thickness (by micrometer, part of EQM—Egg Quality Measurement electronic set, TSS), albumen and yolk acidity (by pH meter with a combined glass electrode). The collected eggshells were subjected to a microscopic analysis using a stereoscopic microscope (Olympus SZX16, magnification 8 × ).

### Main Experiment

The material for the main experiment consisted of 780 brown-shelled table hen eggs (Tetra SL, commercial stock, cage system), individually numbered and placed on trays 30 pcs on each. All eggs were collected and subjected to experimental procedure on the same day. The control group (CA0) consisted of eggs not coated with any additional substances. As experimental factor 2 the most effective concentrations of citric acid solution were chosen based on the results of preliminary study. In groups CA10 and CA15, eggshells were sprayed with the aqueous solution of citric acid at 10 and 15% concentration, respectively. The schema of the experiment is presented in Table 1. At the start of the experiment, quality traits of eggs from the control group were analyzed exclusively. The remaining eggs were stored at 14°C and 70% RH (typical storage conditions) and their quality was evaluated after 7, 14, 21, and 28 d.

**Table 1:** Schema of the main experiment (number of eggs).

Time (days)	Treatments		
	Control	Sprayed with citric acid	
		10%	15%
	CA0	CA10	CA15
0	60		
7	60	60	60
14	60	60	60
21	60	60	60
28	60	60	60
Total	360	240	240

The following experimental material characteristics were evaluated:

• whole egg—depth of the air cell (**ACD**, visually using the template), mass (EW, using an electronic scale with an accuracy of 0.01 g), proportions of morphological elements (in relation to egg weight: **YP**, yolk proportion in egg weight; **AP**, albumen proportion in egg weight; SP, shell proportion in egg weight),
• shell—weight (**SW**, using an electronic scale with an accuracy of 0.01 g), thickness (**ST**, by EQM micrometer screw, on the “equator”), strength (**SS**, Instron 55 Mini apparatus, along the long axis, blunt end up),
• albumen—weight (**AW**, using an electronic scale with an accuracy of 0.01 g), height (**AH**, EQM detector), pH (**ApH**, pH meter with a combined glass electrode),
• yolk—weight (**YW**, using an electronic scale with an accuracy of 0.01 g), color (**YC**, using 16-points Roche, DSM), index (**YI**, as ratio of its height and diameter), pH (**YpH**, pH meter with a combined glass electrode).

Based on the obtained data, additional quality parameters were calculated, such as

• weight loss (**WL**), using an electronic scale with an accuracy of 0.01 g,
• specific mass of eggs (**ESG**), as per Archimedes' principle based on egg weight measured in the air (“dry egg weight”) and in the water (“wet egg weight”),
• shell density (**SD**) calculated in accordance with the formula proposed by Shafey (2002),
• water vapor permeability (ESC, egg shell conductance) calculated in accordance with the method proposed by Ar et al. (1974) with adjustment to eggs storage conditions (temperature, humidity), expressed in SI units,
• Haugh units (**HU**, according to Williams, 1992).

The samples collected from equator parts of shells (surface and cross-section) were subjected to a microscopic analysis. Micrographs were taken using a scanning electron microscope FEI QUANTA 200 SEM (Hillsboro, OR) operated at 25 kV.

The obtained data were statistically analyzed using the SPSS 24.0 statistical package (IBM Corp., 2016; IBM Corporation, 2016). The normality of data distribution was tested using Shapiro-Wilk test. The groups in preliminary experiment were compared using the one-way analysis of variance with Tukey's post hoc test at the significance level *P* ≤ 0.05. In the main research, the two-factorial analysis of the model including the influence of time (**T**) and citric acid coverage (**CA**) as well as the interaction between both factors was conducted.

## Results

### Preliminary Experiment

On the basis of the preliminary study (Table 2), no significant differences in egg weight were found regardless of the experimental group. Significant differences were found in the range of egg weight loss in the preliminary study, with the highest value being observed for eggs from the control group and the lowest recorded for 10 and 15% citric acid concentrations. Importantly, eggs coated with 5% citric acid did not differ significantly from those in the control group. A similar trend was observed in the analysis of the change in air cell depth. After 28 d of storage, the highest values, exceeding the limits set by the Commission Regulation (EC) No. 589/2008, were found in the CA0 group. It should also be noted that eggs from groups CA5 and CA20 did not differ significantly from the control group. On the other hand, for concentrations of 10 and 15%, these values were significantly lower.

**Table 2:** The results of the preliminary experiment, characteristics of whole egg depending on the experimental group.

Characteristic	T (days)	CA0	CA5	CA10	CA15	CA20	SEM
EW (g)	0	36.96	36.69	36.85	35.84	37.73	0.295
	7	36.06	36.24	36.22	35.46	37.21	0.283
	14	35.40	35.93	35.73	35.21	36.84	0.277
	21	34.54	35.51	35.12	34.89	36.38	0.275
	28	33.94	35.21	34.70	34.67	36.06	0.278
WL (%)	0-28	8.168^y^	5.013^x,y^	3.559^x^	4.556^x^	4.297^x^	0.397
ACD (mm)	7	3.30^y^	2.56^x,y^	2.13^x^	2.44^x,y^	2.50^x,y^	0.122
	14	4.60	3.75	3.38	3.81	4.11	0.145
	21	6.10^y^	4.94^x,y^	3.81^x^	4.31^x^	4.44^x^	0.183
	28	7.00^y^	5.75^x,y^	4.81^x^	5.19^x^	5.39^x,y^	0.210
SS (N)	28	37.90	41.54	43.19	46.59	41.24	1.817
ST (mm)		0.301	0.291	0.294	0.302	0.287	0.003
ApH		9.35	9.32	9.23	9.33	9.30	0.018
YpH		7.11	7.17	6.95	7.17	6.21	0.214

^x,y^means within the row differ significantly at *P* ≤ 0.05.

Abbreviations: ACD, air cell depth; ApH, albumen pH; CA, citric acid; EW, egg weight; SS, shell strength; ST, shell thickness; WL, weight loss; YpH, yolk pH.

No significant differences in eggshell quality parameters (strength and thickness) were noted. This observation is important because it indicates that there is no negative impact of the applied experimental factor. In addition, changes in the pH of morphological elements indicate only a surface reaction of citric acid, regardless of its concentration.

**Figure 1:**
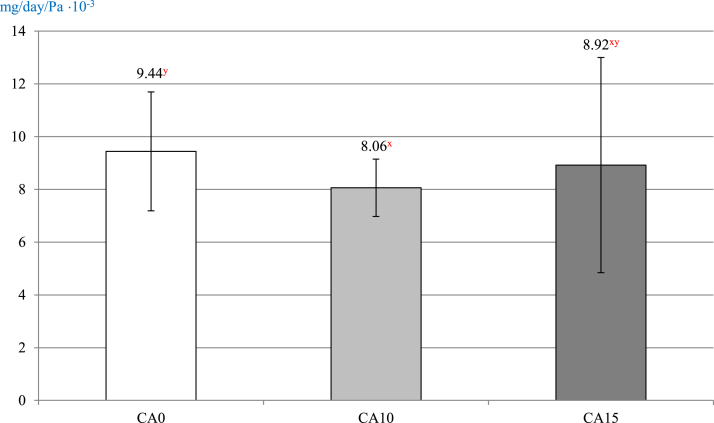
The eggshell permeability after 28 d of storage depending on the specific concentration of citric acid applied on the shell, ^x,y^ means within the row differ significantly at *P* ≤ 0.05.

An additional element of the work involved the photography of shells' surface treated with various concentrations of citric acid with the use of stereoscopic microscope (Figure 1). It was observed that eggs treated with the acid (especially 10 and 15%) had a higher sheen and their surface looked smoother. These observations confirm the occurrence of reactions between shell components (mainly calcium) and the experimental factor. It was also found that the eggshells of CA20 group had marks of minor damage, which may suggest an excessively high concentration of the acid.

The results obtained in the preliminary experiment showed that the most effective concentration of citric acid seems to be 10 or 15%. It does not deteriorate the eggshell quality as well as contributes to egg content preservation.

### Main Experiment

The results concerning the quality characteristics of the whole egg are presented in Table 3. A significant influence of time was found in all groups covered by the experiment. One of the fundamental changes in the quality of eggs during the storage is their weight loss. The first significant differences were observed as early as after 14 d of the experiment, with the highest weight loss being observed in eggs from the control group, and the lowest in those sprayed with 10% citric acid solution. CA15 group did not differ significantly from the other groups included in the experiment. After 28 d, it was found that eggs from the control group were characterized by the highest weight loss. The experimental groups showed significantly lower values of this parameter and did not differ from each other. It should also be noted that WL was influenced by both factors (time and citric acid) as well as their interactions. The ESG was significantly influenced by time and the experimental factor, with no interaction between them; however, no differences were observed between groups at the end of the study.

**Table 3:** Characteristics of the whole egg depending on the specific concentration of citric acid applied on the shell.

Characteristic	Time (days)	Group				SEM	Factors		
		CA0	CA10	CA15	Total		CA	T	CA × T
EW (g)	0	60.76	61.53	60.82	61.04^c^	0.402	1	1	-
	7	59.29	60.88	60.22	60.04^b,c^	0.399			
	14	57.65	59.51	58.73	58.67^a,b^	0.391			
	21	57.07	59.21	58.41	58.56^a^	0.398			
	28	56.53^x^	58.88^y^	58.05^x,y^	58.18^a^	0.397			
ESG (g/cm^3^)	0	1.088			1.088^e^	0.004	1	1	-
	7	1.071^x^	1.079^y^	1.079^y^	1.076^d^	0.001			
	14	1.069	1.073	1.074	1.072^c^	0.001			
	21	1.058^x^	1.066^y^	1.070^y^	1.064^b^	0.001			
	28	1.054	1.055	1.052	1.054^a^	0.001			
WL (%)	0–7	1.085	1.019	0.991	1.032^b^	0.026	1	1	1
	7–14	2.774^y^	2.247^x^	2.559^x,y^	2.527^c^	0.075			
	14–21	1.012^y^	0.512^x^	0.477^x^	0.657^a^	0.069			
	21–28	0.952^y^	0.558^x^	0.625^x^	0.712^a^	0.033			
	0–28	5.703^y^	4.278^x^	4.551^x^	4.844	0.126			
ACD (mm)	7	2.683^z^	1.450^x^	1.733^y^	1.955^a^	0.086	1	1	1
	14	4.093^y^	2.383^x^	2.517^x^	2.998^b^	0.115			
	21	4.567^y^	3.650^x^	3.383^x^	3.867^c^	0.102			
	28	4.700	4.400	4.550	4.517^d^	0.082			

^x,y^means within the row differ significantly at *P* ≤ 0.05; ^a,b^ means within the column differ significantly at *P* ≤ 0.05.

Abbreviations: ACD, air cell depth; CA, citric acid; ESG, egg specific gravity; EW, egg weight; T, time; WL, weight loss.

1 The influence of factor significant at *P* ≤ 0.05.

The air cell depth changed during egg storage. The first time-specific differences between the groups were observed after merely 7 d of the trial. On both the 14th and 21st day of storage, the eggs from the CA0 group were characterized by significantly deeper air cell compared with both experimental groups. Interestingly, no significant differences between the groups were found after 28 d of storage. As in the case of WL, a significant influence of both experimental factors and their interactions was found.

Eggshell permeability was also analyzed (Figure 1). It was found that the highest value of this parameter characterized eggs from the control group (*P* = 0.032), with significantly lower values for CA10 and CA15 groups.

The results of the other eggshell quality analysis are presented in Table 4. The experiment revealed that the application of citric acid did not deteriorate the shell's strength. Moreover, after 7 d of storage, the strength of eggshells from groups CA10 and CA15 was significantly improved in comparison with those from group CA0. However, these observations were not confirmed at the end of the trial (after 28 d).

**Table 4:** Characteristics of the eggshells depending on the specific concentration of citric acid applied on the shell.

Characteristic	Time (days)	Group				SEM	Factors		
		CA0	CA10	CA15	Total		CA	T	CA × T
SS (N)	0	58.54			58.54^a^	2.516	-	1	1
	7	53.65^x^	61.82^y^	61.10^y^	58.86^a,b^	1.116			
	14	60.35	61.88	62.03	61.42^b^	0.469			
	21	61.18	62.37	61.27	61.61^b^	0.438			
	28	59.55	56.63	58.33	58.17^a^	1.520			
SW (g)	0	8.193			8.193^b^	0.152	-	-	-
	7	7.750	7.987	7.913	7.883^a,b^	0.078			
	14	7.611	7.817	7.793	7.740^a^	0.113			
	21	7.898	7.938	7.947	7.928^a,b^	0.083			
	28	7.805	7.830	7.693	7.776^a,b^	0.070			
SP (%)	0	13.48			13.48^b^	0.186	-	1	-
	7	12.83	13.04	13.11	12.99^a,b^	0.117			
	14	12.69	13.03	12.90	12.87^a^	0.169			
	21	13.29	13.17	13.40	13.29^a,b^	0.093			
	28	13.30	13.20	13.20	13.23^a,b^	0.086			
ST (mm)	0	0.332			0.332^b,c^	0.008	1	1	1
	7	0.329^x^	0.336^x,y^	0.343^y^	0.336^b,c^	0.005			
	14	0.324^x^	0.361^y^	0.357^y^	0.347^c^	0.004			
	21	0.282^x^	0.303^x,y^	0.322^y^	0.302^a^	0.005			
	28	0.323^y^	0.326^y^	0.299^x^	0.316^a,b^	0.003			
SD (g/cm^3^)	0	3.41			3.41^b^	0.097	1	1	1
	7	3.46^y^	3.24^x^	3.19^x^	3.30^a,b^	0.031			
	14	3.20	2.99	3.01	3.07^a^	0.048			
	21	4.14^y^	3.69^x,y^	3.47^x^	3.78^c^	0.086			
	28	3.40^x^	3.35^x^	3.63^y^	3.46^b,c^	0.035			

^x,y^means within the row differ significantly at *P* ≤ 0.05; ^a,b^ means within the column differ significantly at *P* ≤ 0.05.

Abbreviations: CA, citric acid; SD, shell density; SP, shell proportion in egg weight; SS, shell strength; ST, shell thickness; SW, shell weight; T, time.

1 The influence of factor significant at *P* ≤ 0.05.

No significant differences were observed in the weight of the eggshell, as well as its proportion in the total egg weight, regardless whether citric acid was used or concerning its specific concentration.

The thickness and density of the shell was observed to be significantly influenced by experimental factors and their interactions (*P* = 0.005). It was found that citric acid at a concentration of 15% reduced the thickness of the shell while increasing its density comparing to both other groups.

Storage time significantly affected all the characteristics of albumen quality (Table 5). The albumen height differed significantly between treatments after 21 d of egg storage. The highest albumen was noted in eggs from CA10 group, but it did not differ from the control one. Lower values were recorded for the CA15 group. This relation was not confirmed after 28 d of egg storage. Taking into account the relation between albumen height and Haugh units, the same effect as observed for Haugh's unit.

**Table 5:** Characteristics of the egg albumen depending on the specific concentration of citric acid applied on the shell.

Characteristic	Time (days)	Group				SEM	Factors		
		CA0	CA10	CA15	Total		CA	T	CA × T
AH (mm)	0	8.22			8.22^c^	0.198	-	1	-
	7	5.69	5.44	5.74	5.62^b^	1.433			
	14	4.94	4.90	5.48	5.11^b^	0.110			
	21	4.52^y^	4.63^y^	4.37^x^	4.51^a^	0.094			
	28	4.11	4.22	4.11	4.15^a^	0.095			
HU	0	90.9			90.9^c^	1.174	-	1	-
	7	73.4	70.8	74.2	72.8^b^	0.827			
	14	67.5	66.7	71.3	68.5^b^	0.948			
	21	64.2^y^	65.7^y^	62.1^x^	64.0^a^	0.973			
	28	60.3	64.2	59.2	61.2^a^	1.593			
AW (g)	0	37.27			37.27^b^	0.734	-	1	-
	7	35.98	36.47	35.88	36.11^a,b^	0.373			
	14	36.45	36.40	36.06	36.30^a,b^	0.405			
	21	35.26	35.22	34.78	35.09^a^	0.336			
	28	34.89	34.86	34.87	34.87^a^	0.378			
AP (%)	0	61.15			61.15^b^	0.531	-	1	-
	7	59.47	59.34	59.19	59.33^a^	0.285			
	14	59.9	60.53	59.59	60.01^a,b^	0.367			
	21	59.81	58.87	59.61	59.43^a^	0.392			
	28	58.79	58.32	58.62	58.58^a^	0.265			
ApH	0	8.25			8.25^a^	0.039	-	1	1
	7	8.92	9.02	8.97	8.97^b^	0.020			
	14	9.09	9.03	9.02	9.05^b^	0.014			
	21	9.09	9.09	9.09	9.09^b^	0.006			
	28	9.11	9.09	9.11	9.10^b^	0.006			

^x, y^means within the row differ significantly at *P* ≤ 0.05; ^a, b^ means within the column differ significantly at *P* ≤ 0.05.

Abbreviations: CA, citric acid; AH, albumen height; AP, albumen proportion in egg weight; ApH, pH of albumen; AW, albumen weight; T, time.

1 The influence of factor significant at *P* ≤ 0.05.

A significant effect of storage time was also proved by the range of albumen weight and its percentage proportion in the egg weight. However, no variability between experimental groups was found within the individual time points.

Groups did not differ significantly with respect to changes in the albumen pH during storage. However, a significant influence of time and interactions of experimental factors on changes in the albumen pH was observed.

Similarly as in the case of egg albumen, the quality characteristics of yolk (Table 6) were also determined by storage time. However, no significant differences were noted in its weight or percentage share in the egg weight regardless of the experimental group.

**Table 6:** Characteristics of the egg yolk depending on the specific concentration of citric acid applied on the shell.

Characteristic	Time (days)	Group				SEM	Factors		
		CA0	CA10	CA15	Total		CA	T	CA × T
YW (g)	0	15.37			15.37^a^	0.267	-	1	-
	7	15.88	15.74	15.70	15.77^b^	0.141			
	14	16.65	16.42	16.55	16.54^a,b^	0.160			
	21	16.87	16.50	16.59	16.65^b^	0.153			
	28	17.13	16.72	16.72	16.86^a,b^	0.272			
YP (%)	0	25.37			25.37^a^	0.525	-	1	-
	7	27.70	27.62	27.70	27.67^b^	0.246			
	14	27.41	26.44	27.51	27.12^b^	0.291			
	21	26.90	27.96	26.99	27.28^b^	0.383			
	28	27.91	28.48	28.20	28.20^b^	0.263			
YI	0	44.47			44.47^c^	0.579	-	1	-
	7	41.91	40.33	42.99	41.74^b^	0.725			
	14	38.11^x^	39.00^y^	40.18^y^	39.10^b^	0.373			
	21	37.53^x^	37.52^x^	38.37^y^	37.81^b^	0.376			
	28	33.99^x^	36.53^y^	36.02^y^	35.51^a^	0.334			
YC (pts)	0	13.48			13.84^b^	0.162	-	1	-
	7	12.80	12.20	12.63	12.54^a^	0.122			
	14	12.46	12.14	12.30	12.30^a^	0.088			
	21	12.53	12.55	12.23	12.44^a^	0.087			
	28	12.50^y^	12.13^x,y^	11.83^x^	12.15^a^	0.107			
YpH	0	6.29			6.29^a^	0.013	-	1	-
	7	6.37^y^	6.35^y^	6.26^x^	6.33^a,b^	0.013			
	14	6.38	6.36	6.39	6.38^b^	0.013			
	21	6.42^x^	6.58^y^	6.46^x^	6.49^c^	0.017			
	28	6.54	6.48	6.51	6.51^c^	0.021			

^x,y^means within the row differ significantly at *P* ≤ 0.05; ^a,b^ means within the column differ significantly at *P* ≤ 0.05.

Abbreviations: CA, citric acid; T, time; YC, yolk color; YI, yolk index; YP, yolk proportion in egg; YpH, pH of yolk; YW, yolk weight.

1 The influence of factor significant at *P* ≤ 0.05.

Significant differences in the yolk shape index were demonstrated as early as after 14 d of the experiment. Higher values of this parameter characterized eggs treated with citric acid. On the 21st day of the experiment, no differences between CA0 and CA10 groups were registered. However, after 28 d of storage, differences between the control and experimental groups were statistically significant with higher YI values for the latter ones.

As far as the yolk color is concerned, significant differences between the groups were noted. The significantly darkest yolk color after 28 d of egg storage characterized eggs from group CA0, whereas the brightest one was recorded for eggs from CA15 group.

The yolk pH was determined by the time of egg storage. However, significant differences between the experimental groups were recorded exclusively after 7 and 21 d of storage. Eggs from CA15 group were characterized by lower pH, with higher values recorded for CA10 group.

**Figure 2:**
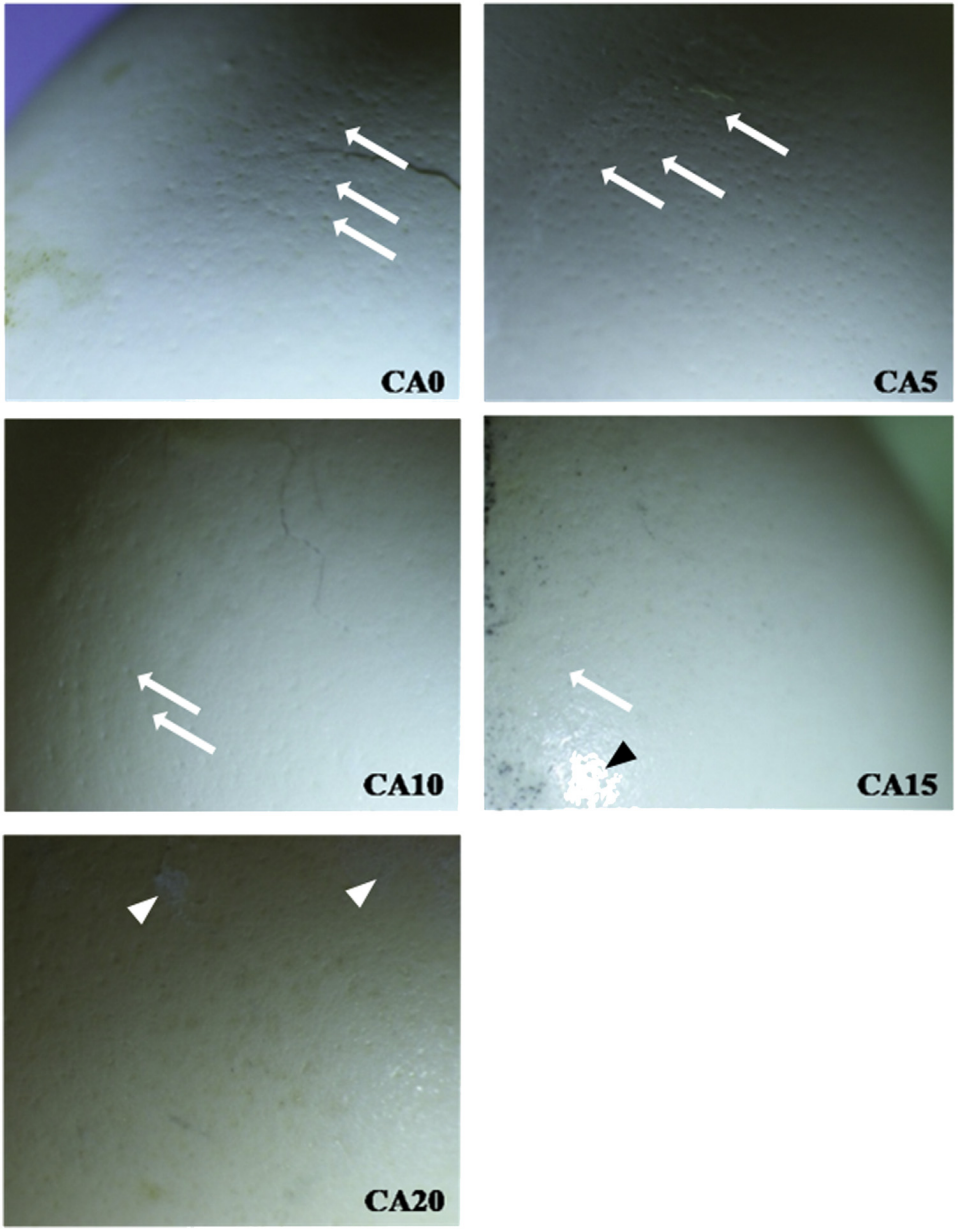
The effect of various concentrations of citric acid on the eggshell surface (magnification 8 × ). CA0, control group; CA5, CA10, CA15, CA20, the concentration of citric acid at 5, 10, 15, and 20%, respectively; CA0 and CA5, well visible shell pores without visible changes (white arrows); CA10, pores are shallower (white arrows); CA15, almost no visible pores (white arrow) and visible smoothing of the shell surface (black arrow head); CA20, the citric acid salts visible as the form of white powder (white arrow heads).

**Figure 3:**
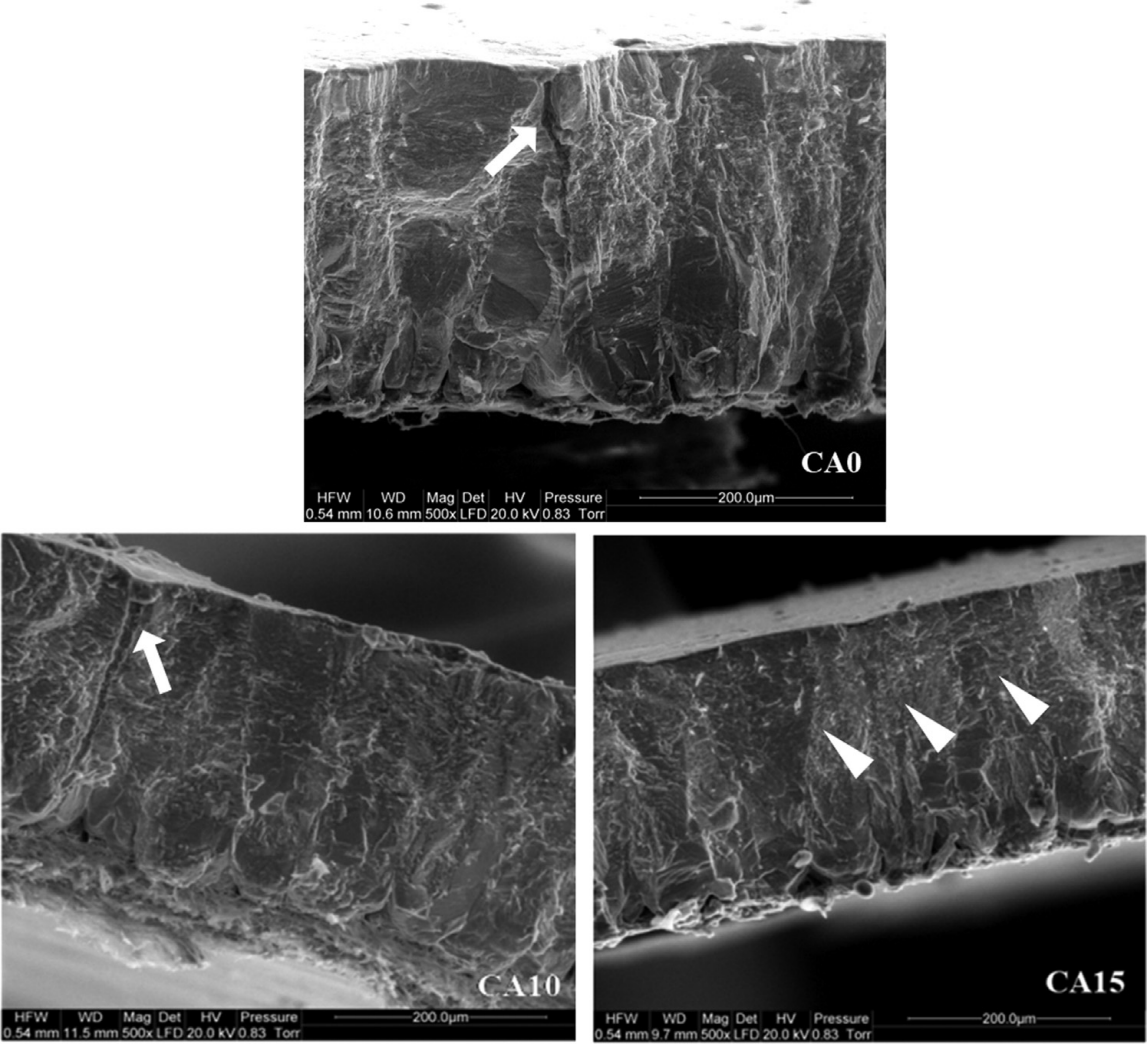
The effect of various concentrations of citric acid on the eggshell surface (scanning microscope). CA0, control group; CA10, CA15, the concentration of citric acid at 10 and 15%, respectively; CA0, the permeable eggshell pore visible in cross-section (white arrow); CA10, the eggshell pore sealed at the external surface due to the effect of citric acid (white arrow); CA15, visible changes in the integrity of the eggshell (white arrow heads).

The scanning electron microscopy technique (Figure 2) revealed that pores were closed in eggshells treated with citric acid. In addition, in the case of eggshells from CA15 group, a higher integrity of the surface layer was found. Figure 3 shows the effect of various concentrations of citric acid on the eggshell surface and its cross-section. On the CA0 micrograph, the permeable eggshell pore is visible in the cross-section. The CA10 demonstrates the eggshell pore sealed at the external surface due to the effect of citric acid, whereas CA15, the changes in eggshell integrity.

## Discussion

Storage time is one of the fundamental factors affecting the eggs' quality deterioration (Samli et al., 2005; Brodacki et al., 2019). One of the main changes is the loss of egg weight due to water evaporation. Therefore, the reduction of gas exchange (water evaporation) allows the process of the eggs' “aging” to be inhibited. This is confirmed by the results of own research and data presented by other authors (Caner, 2005; Jo et al., 2011). Pires et al. (2019) demonstrated that eggshells coating with substances of various origin (mineral oil or rice protein) reduces the weight loss. In addition, authors showed that the effectiveness of rice protein depended on its dose. The type of the substance used was an important inhibitor in this respect. The data presented by Caner and Yüceer (2015) showed significant differences in egg weight loss during 28 d of storage depending on the substance such as whey protein isolate, whey protein concentrate, zein and shellac. The best results were obtained after the use of shellac. However, it should be noted that these substances covered only the surface of the eggshell, while in own research the experimental agent reacted with it. Owing to the high calcium content in the eggshell, citric acid reacted with it giving citrates. This led to a change in the outer structure of the eggshell and to seal off the shell pores with citric acid salts.

The egg weight loss during storage also affects the depth of the air cell. In previous studies (Drabik et al., 2018), as well as in this research, a positive effect of coating eggshells with preserving agents was found to limit the air cell deepening during eggs' storage. This dependence is directly related to the water permeability of the shell, which both in our own research as well as in the aforementioned study was reduced by shells being coated with substances that seal the pores.The eggshell has a natural mucine layer to protect the eggs from microbial penetration (De Reu et al., 2006), but as the time passes from egg laying, the eggshell pores become unsealed (Rodrigez - Navarro et al., 2013). At the same time, studies show that the egg washing procedure limited the thickness of the mucin layer, which, however, did not significantly affect the depth of the air chamber during egg storage (Liu et al., 2016). The Rodrigez - Navarro et al. (2013) study showed that eggshell cuticle is composed of 2 basic layers: the outer layer, rich in proteins, and the inner one, made of sulfated polysaccharides and phosphates. Citric acid, as a weak organic acid, should not react with any of the cuticle components, even in relatively high concentrations like those used in our study. It can therefore be assumed that the action of the acid focused on the inorganic eggshell layer, whereas the mucine layer was only affected to the extent resulting from the test substance application procedure (spraying).

Although the substance used reacts with shell-building elements, no significant quality deterioration was found. In terms of the shell quality, one the most important features is its strength. Jones and Musgrove (2005) demonstrated that storage time does not significantly affect the shell strength, which was also confirmed by own research. However, it should be noted that certain methods of limiting changes in the eggs' quality during the storage may have a negative impact on this parameter. For example, it has been shown that the use of vacuum packing (Aygun and Sert, 2013) can cause shell cracking, while some of the substances increased shells' strength (Caner and Canzis, 2008; Biladeau and Keener, 2009). In the case of own research, the absence of significant differences between the groups is a positive effect as it confirms the surface action of citric acid without damaging the shell structure.

Storage time of table eggs strongly affects the albumen quality, which is confirmed by studies by Samli et al. (2005), Brodacki et al. (2019), as well as by own research. One of the fundamental qualitative changes during the storage of table eggs is a decrease in the height of dense albumen and the associated number of Haugh units. Studies have shown a relationship between ovomucin and albumen height, which changes with storage time. In accordance with the data presented by Wang et al. (2019), there is a negative correlation between the content of ovomucin and pH. Considering the above, it may be concluded that changes in the albumen alkalinity will significantly reduce the value of other egg quality assessment parameters. Alkalization of albumen is a natural phenomenon associated with the release of carbon dioxide from egg content (Monira et al., 2003). Therefore, the use of sealing compounds allows limiting the loss of CO_2_ from the egg content, which contributes to maintaining a higher albumen quality. This relationship is confirmed by numerous works (Biladeau and Keener, 2009; Pires et al., 2020), as well as by own research, where significant differences in albumen height and Haugh units after 21 days of storage were noted. At the same time, it should be noted that the use of citric acid had no direct effect on the albumen pH at the beginning of the experiment, which only confirms its surface effect. The subsequent variability is the effect of limiting the possibility of releasing carbon dioxide from the egg content by limiting the permeability of the shell pores.

During egg storage, the weight of yolk increases because of the diffusion of water from albumen to yolk (Menezes et al., 2012). This process leads additionally to the change of yolk color and shape index. In our own research, as well as in studies presented by other authors using chitosan, whey protein concentrate, soybean oil (Wardy et al., 2010), and propolis (Copur et al., 2008), an increase in yolk mass during egg storage was found. It is noteworthy that the use of a protective factor inhibited the increase of this characteristic. The yolk shape index was also reduced. The use of citric acid allowed inhibiting this process, which is indicated by significantly higher values after 28 d of storage in the CA10 and CA15 groups. A similar effect can be obtained using other shell coating substances such as chitosan in conjunction with organic acids (Caner and Cansiz, 2007) or soybean protein isolate in conjunction with montmorillonite (Xu et al., 2017). Similarly to changes in albumen quality, the traits of yolk changed not directly as a result of citric acid, but through its effect on the permeability of the eggshell pores. Limiting weight loss, release of carbon dioxide and water vapor from the egg content also contributed to limiting changes of yolk traits, so citric acid inhibited changes in the quality of this egg element only indirectly through direct action on the eggshells.

## Conclusion

The use of citric acid led to a reduction of qualitative changes in eggs demonstrated by reducing the weight loss, shallower air cell, higher structural albumen, less-intensive water diffusion from albumen to yolk indicating the improved resistance of vitelline membrane.

Owing to the fact that citric acid is accepted and recognized as a safe preservative and is a relatively cheap and available substance, it seems that it can be used to limit the quality changes in table eggs during their storage.

Because of the protective effect and the lack of damage signs of the eggshell surface, it seems that the recommended concentration of citric acid used as a coating factor during eggs storage may be 10%.
